# Avoidance of Protein Fold Disruption in Natural Virus Recombinants

**DOI:** 10.1371/journal.ppat.0030181

**Published:** 2007-11-30

**Authors:** Pierre Lefeuvre, Jean-Michel Lett, Bernard Reynaud, Darren P Martin

**Affiliations:** 1 CIRAD, UMR 53 PVBMT CIRAD-Université de la Réunion, Pôle de Protection des Plantes, Ligne Paradis, Saint Pierre, La Réunion, France; 2 Institute of Infectious Diseases and Molecular Medicine, University of Cape Town, Observatory, South Africa; The Pennsylvania State University, United States of America

## Abstract

With the development of reliable recombination detection tools and an increasing number of available genome sequences, many studies have reported evidence of recombination in a wide range of virus genera. Recombination is apparently a major mechanism in virus evolution, allowing viruses to evolve more quickly by providing immediate direct access to many more areas of a sequence space than are accessible by mutation alone. Recombination has been widely described amongst the insect-transmitted plant viruses in the genus *Begomovirus* (family Geminiviridae), with potential recombination hot- and cold-spots also having been identified. Nevertheless, because very little is understood about either the biochemical predispositions of different genomic regions to recombine or what makes some recombinants more viable than others, the sources of the evolutionary and biochemical forces shaping distinctive recombination patterns observed in nature remain obscure. Here we present a detailed analysis of unique recombination events detectable in the DNA-A and DNA-A-like genome components of bipartite and monopartite begomoviruses. We demonstrate both that recombination breakpoint hot- and cold-spots are conserved between the two groups of viruses, and that patterns of sequence exchange amongst the genomes are obviously non-random. Using a computational technique designed to predict structural perturbations in chimaeric proteins, we demonstrate that observed recombination events tend to be less disruptive than sets of simulated ones. Purifying selection acting against natural recombinants expressing improperly folded chimaeric proteins is therefore a major determinant of natural recombination patterns in begomoviruses.

## Author Summary

The exchange of genetic material between different virus species, called inter-species recombination, has the potential to generate, within a single genome replication cycle, an almost unimaginable number of genetically distinct virus strains, including many that might cause deadly new human, animal, or plant diseases. Many fear that inter-species recombination could provide viruses with quick access to evolutionary innovations such as broader host ranges, altered tissue tropisms, or increased severities. However, mounting evidence suggests that recombination is not an unconstrained process and that most inter-species recombinants that occur in nature are probably defective. It is suspected that networks of coevolved interactions between different parts of virus genomes and their encoded proteins must be kept intact for newly formed inter-species recombinants to have any chance of out-competing their parents. One category of coevolved interaction is that between contacting amino acids within the 3-D structures of folded proteins. Here we examine the distributions of recombination events across the genomes of a group of rampantly recombining plant viruses and find very good evidence that this class of interaction tends to be preserved amongst recombinant sequences sampled from nature. This indicates that selection against misfolded proteins strongly influences the survival of natural recombinants.

## Introduction

Besides its vital cellular role in maintaining and repairing broken DNA molecules [1,2], recombination is also evolutionarily significant in that it defends genomes against the otherwise unavoidable accumulation of deleterious mutations [3–5]. However, by enabling the creation of novel genetic combinations from existing genomes, recombination has the potential to do more than just reverse the mutational decay of genomes: it can also provide organisms with vastly more evolutionary options than are available through mutation alone [6,7].

In virology, two recombinational processes can be distinguished: genome reassortment and true recombination. Genome reassortment, also called pseudo-recombination, involves the exchange of intact genome components between viruses with multipartite genomes to yield viruses whose genomes are comprised of new combinations of components. True recombination, on the other hand, involves the exchange of genetic material between individual genomic molecules. The rearrangement of genetic information mediated by both true recombination and pseudo-recombination must yield fully functional and reasonably fit genomes for these processes to be easily detectable in nature. However, analysis of the functionality of recombinant genes [8,9] and the viability of recombinant genomes [10–12] has indicated that a large proportion (and possibly the vast majority) of recombination events between genomes sharing less than 90% nucleotide sequence identity yield progeny with decreased viability. Bacterial recombination [13] and DNA shuffling studies [8,9,14,15] have indicated that the evolutionary value of recombination can vary depending on both the specific genes and sub-gene modules that are exchanged. A key factor determining the survival of recombinants is the degree to which recombination disrupts coevolved intra-genome interactions. At the whole genome scale, potentially disrupted interactions could include sequence-specific interactions between viral proteins, DNA, and RNA. At the scale of individual viral proteins, interactions include those occurring between amino acids required for proper folding.

While full accounts of experimentally verified intra-genome interactions are currently unavailable for any virus species, potential amino acid interactions within folded proteins can be inferred with reasonable accuracy given high resolution protein structural data. In the past five years, protein engineers have made substantial progress in the development of computational methods capable of accurately inferring degrees of recombination-induced fold disruption in experimentally generated chimaeras of proteins with known structures [8,14,15]. Although these methods have, to our knowledge, never been used to analyse any naturally generated chimaeric proteins, we realised they should also be useful for understanding breakpoint distribution patterns found within coding regions of recombining virus genomes.

Recently, Lefeuvre et al. [16] reported the first statistically supported evidence of recombination hot- and cold-spots in the genomes of begomoviruses, members of a highly recombinagenic family of single-stranded DNA viruses called the Geminiviridae. Importantly, they detected a substantial number of recombination events within a portion of the begomovirus replication–associated protein (*rep*) gene encoding a protein for which a high resolution crystal structure is available. In this paper we describe an expanded analysis of recombination amongst begomoviruses. We identify sets of unambiguously unique recombination events detectable in publicly available monopartite begomovirus DNA-A-like sequences and bipartite begomovirus DNA-A sequences. We then determine the distribution of recombination breakpoints across the analysed sequences and confirm the recombination hot- and cold-spots identified previously. We use a method called SCHEMA [8] to predict degrees of fold disruption in chimaeric begomovirus Rep and coat protein (CP) molecules (for which a reasonably high resolution structural model exists) expressed by viruses determined to have recombinant *rep* and *cp* genes. We then compare these predictions with those for an exhaustive set of all possible recombination breakpoint pairs within these genes and provide the first statistical evidence to our knowledge that avoidance of protein fold disruption is a major factor shaping the patterns of recombination that are detectable in natural virus populations.

## Results/Discussion

We anticipated that general rules governing the evolutionary advancement of viruses through recombination should be most manifest in virus groups in which distinctive conserved patterns of recombination have emerged [16–21]. Given that begomoviruses are both highly recombinogenic [22] and display some evidence of recombination breakpoint hot- and cold-spots [16], we undertook a detailed analysis of recombination in this group.

### Are Patterns of Recombination Conserved amongst All Begomoviruses?

We began by precisely mapping the distributions of recombination events across begomovirus DNA-A and DNA-A-like sequences sampled throughout the world. Using a battery of recombination signal detection tools and rigorous manual and automated evaluation of recombination signals, we identified sets of 120 and 164 non-ambiguous unique recombination events in the bipartite begomovirus DNA-A and monopartite DNA-A-like sequences, respectively (see Datasets S1 and S2, Figure S1, and Tables S1 and S2 for detailed descriptions of all the detected events).

**Figure 1:**
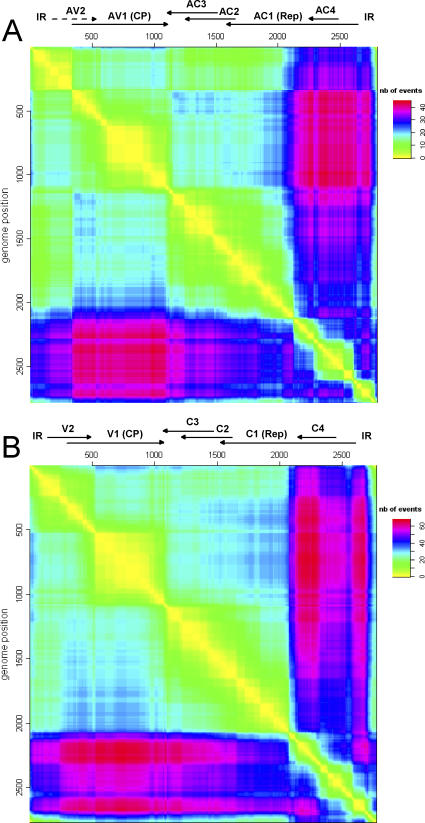
Recombination Region Count Matrix of Unique Recombination Events Detected amongst (A) DNA-A Sequences of Bipartite Begomoviruses and (B) DNA-A-Like Sequences of Monopartite Begomoviruses Unique recombination events have been mapped onto the matrix based on their estimated breakpoint positions. The shades displayed are a function of the number of times pairs of nucleotides (plotted on the *x*- and *y*-axis) are separated during the observed set of unique recombination events. Diagrams indicating the positions of landmarks in begomovirus DNA-A/DNA-A-like sequences are shown on the top of the matrices. Positions were drawn in relative to EACMCV-[TZ] (AY795983) for bipartite sequences and ToLCYT-[Dem] (AJ865341) for monopartite sequences.

These events were mapped onto “recombination count matrices” (Figure 1). These matrices represent the number of times that recombinational movement of sequence tracts within the analysed genomes has separated pairs of nucleotide sites. This representation of the characterised recombination events highlights the differential “exchangeability” of sequence tracts within begomovirus DNA-A and DNA-A-like sequences. Whereas highly exchangeable genome regions (i.e., those separated many times by recombination from their original genetic background) are represented by red/purple shades, the least exchangeable regions (i.e., those separated the fewest times by recombination) are represented by yellow/green shades. As can be seen from Figure 1, the region of the *rep* gene encoding the N-terminal portion of Rep and the adjacent intergenic region sequences up to the virion strand origin of replication (v-*ori*) are the regions of both monopartite and bipartite begomovirus genomes most frequently exchanged during recombination. As a result of this, the 5′ and 3′ portions of *rep* are very frequently inherited from different parents. This implies that *rep* must be comprised of highly modular subregions capable of proper functioning in diverse foreign genetic backgrounds. Conversely, the small numbers of detectable recombination events that separate fragments of the *cp* gene indicate that naturally occurring monopartite and bipartite begomovirus recombinants tend to inherit the portions of this gene encoding CP amino acids 75 through 220 from a single source.

**Figure 2:**
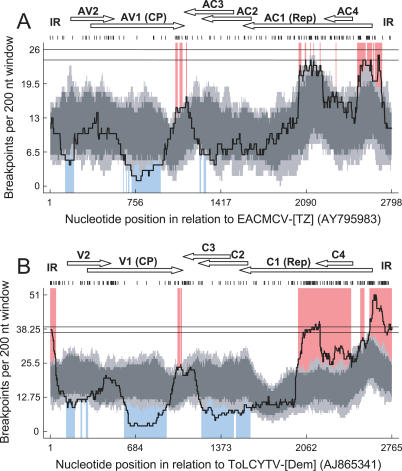
The Distribution of Recombination Breakpoints Detected within (A) DNA-A Sequences of Bipartite Begomovirus and (B) DNA-A-Like Sequences of Monopartite Begomoviruses All estimated breakpoint positions are indicated by small vertical lines at the top of the graph. A 200-nucleotide window was moved along the alignment one nucleotide at a time and the number of breakpoints detected within the window region was counted and plotted (solid line). The horizontal lines at the top of each graph indicate 99% and 95% confidence thresholds for globally significant breakpoint clusters. Light and dark grey areas respectively indicate local 99% and 95% breakpoint clustering thresholds, taking into account local regional differences in sequence diversity that influence the ability of different recombination detection methods to identify recombination breakpoints. Red areas indicate recombination hot-spots, while blue areas represent recombination cold-spots. ORFs (horizontal arrows) and IR are represented on the top of the graph.

To visualise the distribution of recombination breakpoints in monopartite and bipartite begomovirus genomes, all approximated recombination breakpoint locations were plotted on density maps and a permutation test was used to determine whether there were any statistically significant hot- or cold-spots in the breakpoint distribution. This test indicated that the distribution of breakpoints was significantly non-random, with clear recombination hot- and cold-spots being detectable (Figure 2). It is apparent that, as for the recombinant region count matrices (Figure 1), the recombination breakpoint distributions detected in the monopartite and bipartite datasets are very similar. The clusters of inferred breakpoint positions in the two datasets do not, however, have identical significance levels, probably due to differences in datasets with respect to both their sequence diversity, and the number of detectable recombination events they contain.

In both the monopartite and bipartite datasets, larger “globally” significant (global *p*-values <0.05 across its length) and smaller “locally” significant (local *p*-values <0.01) recombination hot-spots are apparent in the intergenic region (IR) and complementary strand ORFs. In the monopartite dataset one large globally significant (*p* < 0.01) hot-spot encompasses the entire IR 5′ of the v-*ori*, and another (*p* < 0.01) occurs near the centre of *rep*. Globally significant hotspots (*p* < 0.05 and *p* = 0.05) are also detected in these positions in the bipartite dataset, but the extent of the IR hot-spot is not as great. This is probably due in large part to the particularly low quality of nucleotide sequence alignment achievable amongst the highly divergent bipartite begomovirus IR sequences. Locally significant hot-spots occur at the interface of *cp* and the C3 ORFs in both bipartite and monopartite sequences. In addition to hot-spots, the analysis also revealed locally significant recombination cold-spots. These occurred in the first half of *cp* and in the third quarter of the V1 ORF for both datasets and in the overlapping region of the C2 and C3 ORFs of the bipartite dataset.

These results clearly indicate that recombination hot- and cold-spots previously identified amongst African and Mediterranean begomoviruses [16] are conserved amongst both monopartite and bipartite begomoviruses found worldwide.

Importantly, the distribution of these recombination hot- and cold-spots is largely consistent with observations made during experimental analyses of geminivirus recombination [23–25] in which the V1/C3 ORF interface and the v-*ori* have been identified as potential recombination hot-spots. Also, analysis of replicating begomoviral DNA intermediates has revealed a wide distribution of so-called heterogeneous length linear dsDNA forms (hDNA). The ends of these hDNA molecules tend to map most frequently to the v-*ori* and either the AC2/AC3 transcription promoter at the hot-spot we detect in the centre of *rep*, or the C2/C3 terminator at the hot-spot we detect at the V1/C3 ORF interface. It has been convincingly demonstrated that these “broken” replicative intermediates are diverted into the recombination-dependent replication pathway of begomoviruses, which would neatly explain the recombination hot-spots detected in these regions [26].

Furthermore, population genetic analysis of recombination rates in large full genome datasets of very closely related groups of maize streak viruses (a geminivirus species in the genus *Mastrevirus*) and cassava-infecting geminiviruses has indicated that the base biochemical recombination rates in sequences encoding complementary sense genes are probably five to 12 times higher than they are in sequences encoding the virion sense genes [27]. Importantly, these studies also show that greatest changes in recombination rates occur near the V1/C3 ORF interface and the v-*ori*.

That all of these lines of evidence indicate the complementary sense ORFs of geminiviruses are biochemically more predisposed to recombination than their virion sense ORFs strongly suggests that something about the direction of transcription of these ORFs may be responsible for the recombination rate imbalance. For example, it has been proposed that complementary sense gene transcription, which occurs in the opposite direction to virion strand synthesis during rolling circle replication of geminivirus genomes, may be responsible for an increased rate of replication complex displacement during replication of the complementary sense ORFs [16,26]. Completion of replication from partially replicated virion strands would then proceed via the recombination-dependent replication pathway [26] which, in the presence of potential template DNAs with dissimilar sequences, could result in an increased prevalence of detectable recombination events across the complementary sense ORFs.

### Do Recombination Breakpoints “Avoid” Disruption of Protein Folding?

While these mechanistic processes might account for both a general imbalance between recombination rates in the virion and complementary sense ORFs and hot-spots at the V1/C3 ORF interface, the centre of *rep* and the v-*ori*, they cannot completely explain, for example, the apparently conserved breakpoint clusters (albeit not hot-spots) at the 3′ end of the V2 ORF and breakpoint cool/cold-spots in the C2 ORF. We have noted previously [16] that, besides the hot-spot in *rep*, peaks in breakpoint density tend to occur either outside or near the ends of genes, whereas cold-spots tend to occur well within genes. As has been previously suggested in analyses of both virus recombination [28] and DNA shuffling experiments [8], this indicated to us that selection might preferentially favour the survival of recombinants that either do not express chimaeric proteins, or express chimaeric proteins in which recombination has not damaged amino acid interactions required for proper protein folding. We therefore decided to test whether selection against disruption of protein folding might not also be at least partially responsible for some of the conserved breakpoint density peaks and troughs observable in Figure 2.

Recombination events with inferred breakpoint positions within the portions of *rep* and *cp* genes that encode protein fragments with available 3-D structural data were identified. These included 12 and five events in the *cp* genes of the monopartite and bipartite begomoviruses, respectively, and 29 and 19 events in the *rep* genes of the monopartite and bipartite begomoviruses, respectively.

We used the SCHEMA method to predict degrees of fold disruption in the chimaeric Rep and CP molecules expressed by the recombinant viruses we identified. This analysis indicated that the average degree of potential fold disruption in the chimaeric Rep molecules was higher than that in the chimaeric CP molecules (E-values in Table 1). Rather than indicating that recombination should be more tolerable in *cp* than it is in *rep*, this result simply reflects that CP molecules are much more conserved than Rep molecules and that there are consequently fewer potentially disruptive combinations of amino acids. It should also be pointed out that both CP and Rep have overlapping ORFs: V2/AV2 in the case of CP and C4/AC4 in the case of Rep. Whereas in the case of CP the overlap with V2/AV2 involves only approximately 3% (six codons) of the analysed CP region, the overlap of Rep and C4/AC4 involves approximately 58% (67 codons) of the analysed Rep region. It is possible that the evolutionary constraints of overlapping coding regions have made Rep more robust than CP with respect to potentially clashing amino acid interactions within its folded structure. Importantly, by increasing the number of tolerable Rep mutations, the only influence increased folding robustness might have had on our analyses would have been to decrease the power of our tests for preservation of intra-protein interactions.

**Table 1:**
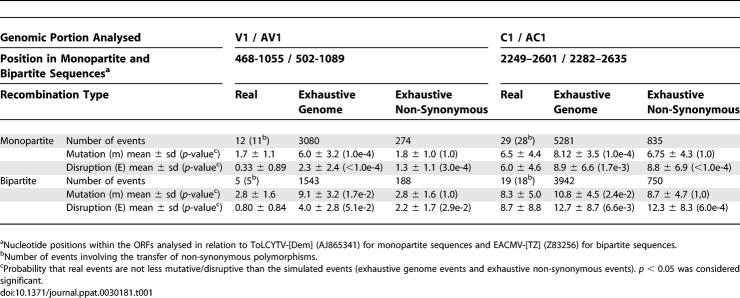
SCHEMA Derived Estimates of Recombination Induced Effective Mutation (m) and Protein Fold Disruption (E) for Monopartite and Bipartite Begomovirus Datasets Comparing Real and Simulated Recombination Events

We performed SCHEMA analyses on the two simulated datasets (described in Materials and Methods) to determine whether the degrees of Rep and CP fold disruption predicted for the observed recombinants were significantly lower than would be expected if selection did not act against recombinants expressing chimaeric proteins with high degrees of predicted fold disruption. Analysis of the exhaustive genome event dataset indicates that predicted degrees of fold disruption in real CP and Rep chimaeras were significantly lower (Table 1; *p*-values = 5.1 × 10^−2^ and < 1.0 × 10^−4^ for the bipartite and monopartite CP datasets, respectively, and 1.7 × 10^−3^ and 6.6 × 10^−3^ for the monopartite and bipartite Rep datasets, respectively) than would be expected in the absence of selection against fold disruption. However, the amino acid mutation levels (m-values in Table 1) of the real datasets indicate that the real recombination events also tended to involve transfers of significantly fewer non-synonymous mutations than the simulated datasets (Table1; *p*-values = 1.0 × 10^−4^ and 1.7 × 10^−2^ for the mono and bipartite CP datasets, respectively, and 1.0 × 10^−4^ and 2.4 × 10^−2^ for the monopartite and bipartite Rep datasets, respectively). This indicated that the significantly reduced degrees of predicted fold disruption in the real datasets relative to the simulated datasets are at least in part due to the real recombination events involving transfers of significantly fewer non-synonymous mutations than the simulated events. This implies that the real breakpoints tend to occur closer to the edges of the analysed regions than one would expect if breakpoints occurred randomly throughout the regions. Given the breakpoint density peaks on either side of the CP encoding V1 ORF (Figure 2), we had anticipated this result for the CP dataset. However, a similar result obtained for the Rep dataset implies that, despite the high density of breakpoints throughout the C1 region encoding the fragment of Rep that was analysed, there is still a significant tendency for breakpoints to occur closer to the edges of the analysed region than would be expected by chance.

We therefore decided to test whether avoidance of protein fold disruption is achieved only through avoidance of non-synonymous mutation mixing, or whether, controlling for unequal degrees of non-synonymous mutation mixing, it is also achieved through preferential mixing of non-disruptive non-synonymous mutations. Analysis of the simulated non-synonymous event dataset indicated that in the natural monopartite and bipartite *cp* and *rep* recombinants, there does indeed appear to have been preferential mixing of non-disruptive non-synonymous mutations (Table 1). This demonstrates, therefore, that whenever an interaction between a pair of polymorphic amino acid residues is predicted to be important for Rep or CP folding, there is a tendency for nucleotide sequences encoding these amino acids to be inherited from the same parental virus significantly more often than those encoding non-interacting pairs of polymorphic amino acids.

Despite the common conception that recombination is a highly efficient mechanism used by both microorganisms and protein engineers in the discovery of phenotypic novelty and/or improved fitness, rules constraining the evolutionary utility of recombination are beginning to emerge. It has been experimentally demonstrated that the viability of recombinant viruses and the activities of chimaeric proteins are strongly influenced by both the relatedness of their parents and the inherent “modularity” of the sequence tracts they inherit from them [8,10]. Put simply, fragments of sequence that do not interact extensively with other sequence fragments tend to function well when transferred into even highly divergent foreign genetic backgrounds, whereas those that interact extensively with other sequence fragments tend to only work properly when transferred into foreign genetic backgrounds that are not very different from those in which they evolved [10,11]. It is therefore probable that high profile natural recombinant viruses, such as those that are responsible for disease outbreaks [29] or those that have novel host ranges and phenotypes [30,31], or even those that have simply emerged as prominent circulating members of virus populations [32–35], represent the exceptional, reasonably fit subset of a vastly greater but vastly more ephemeral group of defective “hopeful monsters” culled by purifying selection.

Our results provide clear supporting evidence for this notion that purifying selection is a major factor shaping at least part of the distinctive patterns of natural recombination found in begomoviruses. Within the genome regions analysed, we find strong statistical evidence that natural recombination events have tended to involve sequence exchanges that avoid the transfer of non-synonymous nucleotide polymorphisms (i.e., those encoding different amino acids in the different parents) between genomes. We also show that, when non-synonymous polymorphisms are transferred between genomes, there is a statistically significant tendency to avoid transfer of those non-synonymous polymorphisms that are predicted to disrupt the folding of expressed chimaeric proteins. While these results indicate that interaction networks required for proper protein folding are preserved in the natural recombinants, they imply that strong selective forces must operate against any novel recombinant in which these interaction networks are not preserved.

A major omission in our analysis of intra-protein amino acid interactions is our failure to consider all the other potential interactions that most likely occur within the genomes analysed. These include many sequence-specific inter-protein and protein–DNA interactions that might also constrain the viability of recombinants [10]. While experimental work towards obtaining high resolution genome-wide interaction maps has only begun for most virus taxa (including the begomoviruses), exciting new analysis methods are being developed to identify both coevolving (or covarying) amino acids within protein sequence alignments [36–38] and epistatically interacting nucleotide sites within DNA sequence alignments [39,40]. Although these methods promise the mapping of interaction networks directly from naturally sampled viral genome sequences, it is currently unknown how well they will fare given datasets containing (1) large numbers of recombinant sequences or (2) obvious recombination hot-spots. It is very likely, for example, that many non-synonymous polymorphisms on sequence tracts between recombination hot-spots will be detectably “covariant” if they are frequently transferred amongst genomes (i.e., on an imposed phylogenetic tree it will appear as though the same sets of sites change simultaneously on multiple branches of the tree). If some of these or future related analytical methods prove robust to the influences of recombination, an obvious application of these would be to determine whether there is also a significant tendency for recombination to avoid disrupting these genome-wide protein–protein, protein–nucleic acid, and nucleic acid–nucleic acid interactions.

In fields as diverse as microbial evolution [41,42], protein engineering [8,9], and computer science [43], maintenance of interaction networks is emerging as a common theme unifying studies aimed at delimiting recombination's potential as an exploratory strategy. Many and complex interactions is a defining feature of living systems. When these interactions are encoded within genome sequences they form an epistatic architecture. It is really just common sense that for productive recombination to occur it must happen without damaging the integrity of these largely intangible network-like structures. Maintenance of these networks might in fact be directly responsible for the evolution of differential biochemical predispositions for recombination across genomes: If recombination events mostly occur in genome regions with low connectivity, a greater proportion of recombinants would be viable than if recombination events were randomly scattered across genome regions with low and high connectivity. Other evolutionary strategies to ensure maintenance of interaction networks in the face of continual recombination might be the evolution of network robustness, or an increased capacity to mutationally compensate for deleterious recombination events. Conversely, however, the network architectures themselves might also evolve over time to accommodate biochemically predisposed recombination hot-spots that have some biological importance. We have shown here that in the case of the begomoviruses at least, various biochemical and selective processes working in tandem most likely combine to produce the distinctive patterns of recombination seen in nature.

## Materials and Methods

### Sequence data.

All available monopartite and bipartite begomovirus DNA-A and DNA-A-like sequences were obtained from public sequence databases using TaxBrowser (http://www.ncbi.nlm.nih.gov/) in May 2006. Multiple sequence alignments were constructed separately for monopartite and bipartite sequences using POA [44], the ClustalW [45] based sub-alignment tool available in MEGA 3.1 [46], and manual editing. While great care was taken to ensure the most accurate alignment possible, during subsequent recombination analyses additional alignment checks were performed in RDP3 (also using the ClustalW method) for every recombination signal detected to ensure that they were not misalignment artefacts [45]. To minimise the number of tests performed during recombination analyses (and therefore increase the statistical power of the analyses) all but one sequence within groups of sequences sharing more than 98% nucleotide identity were discarded. The resulting monopartite DNA-A-like and bipartite DNA-A sequence alignments contained 123 and 116 sequences, respectively.

### Structural data.

The Rep protein structure (catalytic domain; residues 4–121) of TYLCSV has been determined by NMR spectroscopy. This Rep structure (PDB ID 1L2M) comprises five anti-parallel β sheets in the centre with a two-stranded β sheet, a β hairpin, and two α helices on the periphery [47]. The begomovirus capsid structures (196-aa core CP) has been modeled based on the crystal structure of *Satellite Tobacco Necrotic Virus* and fitted into an approximately 20-Å density map generated from cryoelectron microscopy reconstructions of *African cassava mosaic virus* particles [48]. The PDB file of this structure was kindly provided by B. Böttcher.

### Recombination analysis.

Identification of potential recombinants, parental sequences, and approximation of possible recombination breakpoint positions was carried out using the RDP [49], Geneconv [22], RecScan [50], Maximum Chi Square [51], Chimaera [52], and SisterScan [53] methods as implemented in RDP3 [52], which is available from http://darwin.uvigo.es/rdp/rdp.html (for full details of program settings, see Datasets S1 and S2). The analyses were performed with default settings for the detection methods, a Bonferroni-corrected *p*-value cutoff of 0.05, and a requirement that any potential event be detectable by two or more methods. It is important to point out that implementations of all these recombination detection methods in RDP3 were not severely constrained by the initial window size settings specified at the onset of the analyses. All of the methods used include an algorithm for dynamically optimising window sizes for the detection of recombination signals during an initial exploratory phase of recombination detection. Following this exploratory phase, RDP3 rechecks every detected recombination signal with all six methods with a starting window size seeded with that used by whatever method initially detected the signal.

The approximate breakpoint positions and recombinant sequence(s) inferred for every detected potential recombination event were manually checked and adjusted where necessary using the extensive phylogenetic and recombination signal analysis features available in RDP3. This process further reduced any possible influence that initial window size settings had on the final estimates of breakpoint positions. Once a set of unique recombination events was identified, a breakpoint map containing the positions of all clearly identifiable breakpoints was compiled. A breakpoint density plot was then constructed from this map as described in Heath et al. [17].

### SCHEMA analysis.

SCHEMA takes as input a PDB protein structure file and parental amino acid sequence files. It uses the protein structural information to properly fold the parental amino acid sequences and then identifies potentially interacting amino acid pairs based on their proximity (in this case within 4.5 Å) within the resulting folds. The amino acid contact map yielded by this process can be used to determine the degree of fold disruption expected in any conceivable chimaera of the parental amino acid sequences. The way this is done is relatively simple: For all the amino acid residues that are potentially interacting within a folded chimaeric protein, SCHEMA counts the number of instances where the interacting pairs are non-parental. Non-parental interacting amino acid pairs arise when the parental molecules differ from one another at two potentially interacting amino acid residues and the chimaera inherits one-half of the potentially interacting pair from one parent and the other half from the other parent. Counts of these non-interacting pairs in chimaeric proteins, called “E” values, have been shown to correlate directly with degrees of fold disruption experienced by the proteins. The value of E therefore corresponds with expected degree of fold disruption. SCHEMA also counts the number of amino acid substitutions that would be required to convert a chimaera into the parental sequence that it most closely resembles—this value is referred to as “m” [8].

We selected recombination events in the monopartite and bipartite sequence datasets for which (1) sequences closely resembling inferred parental sequences were identifiable and (2) recombination breakpoints occurred in genome regions encoding the portions of Rep and CP with known/approximated 3-D structures. These events constituted a “real event” dataset that we analysed using the SCHEMA method.

We devised a permutation test to determine whether predicted CP and Rep fold disruptions incurred by real events were less severe than those incurred by random recombination events with the same parental sequences simulated throughout the *rep* and *cp* regions under consideration. The permutation test involved two different sets of simulated recombination events. The first set was derived from each real event by moving the breakpoints observed in the real event backwards and forwards along the entire nucleotide sequence alignment one polymorphic alignment position at a time until every possible unique recombination event involving the “exchange” of exactly the same number of polymorphic nucleotides as the real event were simulated within the parental sequences. We called the complete set of simulated events constructed from the entire real event dataset the “exhaustive genome event” dataset. Note that although these events involved exchanges of the same number of total polymorphic sites as was observed for the real events, they can involve a different number of polymorphic sites in the particular genomic regions analysed (i.e., those encoding portions of CP and Rep with available 3-D structure information). This set of simulated events was used to determine whether there was a significant tendency for the observed recombination breakpoints to occur on the edges of these analysed regions. The second set of simulated events was generated by considering only non-synonymous polymorphisms within the alignment regions encoding CP or Rep fragments with available structural data. A window containing the same number of non-synonymous mutations as a corresponding real event was moved along the analysed region, and all possible recombination events were simulated. The subsequent events share exchanges of exactly the same numbers of non-synonymous mutations as the real events and consequently all have the same SCHEMA m values. This set of events was called the “exhaustive non-synonymous event” dataset (see Figure S2 for simulation details). This set of simulated events was used to determine whether, given “exchanges” of the same numbers of polymorphic amino acids as were observed for real events, there was a significant tendency for the real recombination events to exchange less disruptive amino acid polymorphisms.

Quantification of potential fold disruption in real and simulated chimaeric CP and Rep molecules, respectively, expressed by real and simulated recombinants, was carried out using SCHEMA. For each of these chimaeras, amino acid sequences of inferred parents and chimeras were aligned with MUSCLE using default settings [54]. The python scripts SCHEMACONTACTS and SCHEMAENERGY [9] were used to compute *m* (mutational distance between chimaeras and their most closely related parent) and *E* (predicted fold disruption) scores for all simulated and real chimaeras. The analysis procedure was automated using some of the extensive functionalities available in the R package [55], APE [56], and seqinR (available on http://www.cran.r-project.org/). R scripts for these analyses are available on request. We grouped the E and m scores determined for the observed and simulated chimaeras and determined the sum of ranks for the observed chimaeras. We then repeated the entire process 10,000 times but with “real” events randomly chosen from amongst every subset of corresponding simulated events. We propose that the proportion of simulated events with a sum of ranks score lower than or equal to that of the observed event is equivalent to the probability that the breakpoint distributions observed in the real dataset have not tended to avoid disruption of protein folding. Put another way, we estimate a *p*-value from the proportion of permuted recombination event datasets that on the whole are predicted to be less disruptive to protein folding than the set of actual observed recombination events.

## Supporting Information

Dataset S1 RDP3 Bipartite Sequences Analysis RDP3 project file.
(379 KB ZIP)

Dataset S2 RDP3 Monopartite Sequences Analysis RDP3 project file.
(412 KB ZIP)

Figure S1 Recombination Event Density and Parental Sequences Relatedness (25 KB PDF)

Figure S2 Recombination Event Simulation Process Figures describing how the simulated recombination events are created.
(108 KB PDF)

Table S1 Bipartite Sequences Recombination Events List of recombination events detected with RDP3 in bipartite sequences.
(132 KB XLS)

Table S2 Monopartite Sequences Recombination Events List of recombination events detected with RDP3 in monopartite sequences.
(140 KB XLS)

### Accession Numbers

The National Center for Biotechnology Information (http://www.ncbi.nlm.nih.gov/) accession numbers for the sequences discussed in this paper are EACMCV-[TZ] (AY795983), ToLCYTV-[Dem] (AJ865341), and TYLCSV (X61153). The Protein Data Bank (http://www.pdb.org/) ID number for TYLCSV N-terminal region structure is 1L2M.

## Abbreviations

CP: coat protein
hDNA: heterogeneous length linear dsDNA
IR: intergenic region
ORF: open reading frame
Rep: replication-associated protein
v-**ori**;: virion strand origin of replication
