# Supplementary material for: Avoidance of Protein Fold Disruption in Natural Virus Recombinants
Source: PLoS Pathog. 2007 Nov 30;3(11):e181. doi: 10.1371/journal.ppat.0030181 (PMC2092379; doi:10.1371/journal.ppat.0030181)
Supplement: Supplementary file 1 [file ppat.0030181.sg001.pdf]

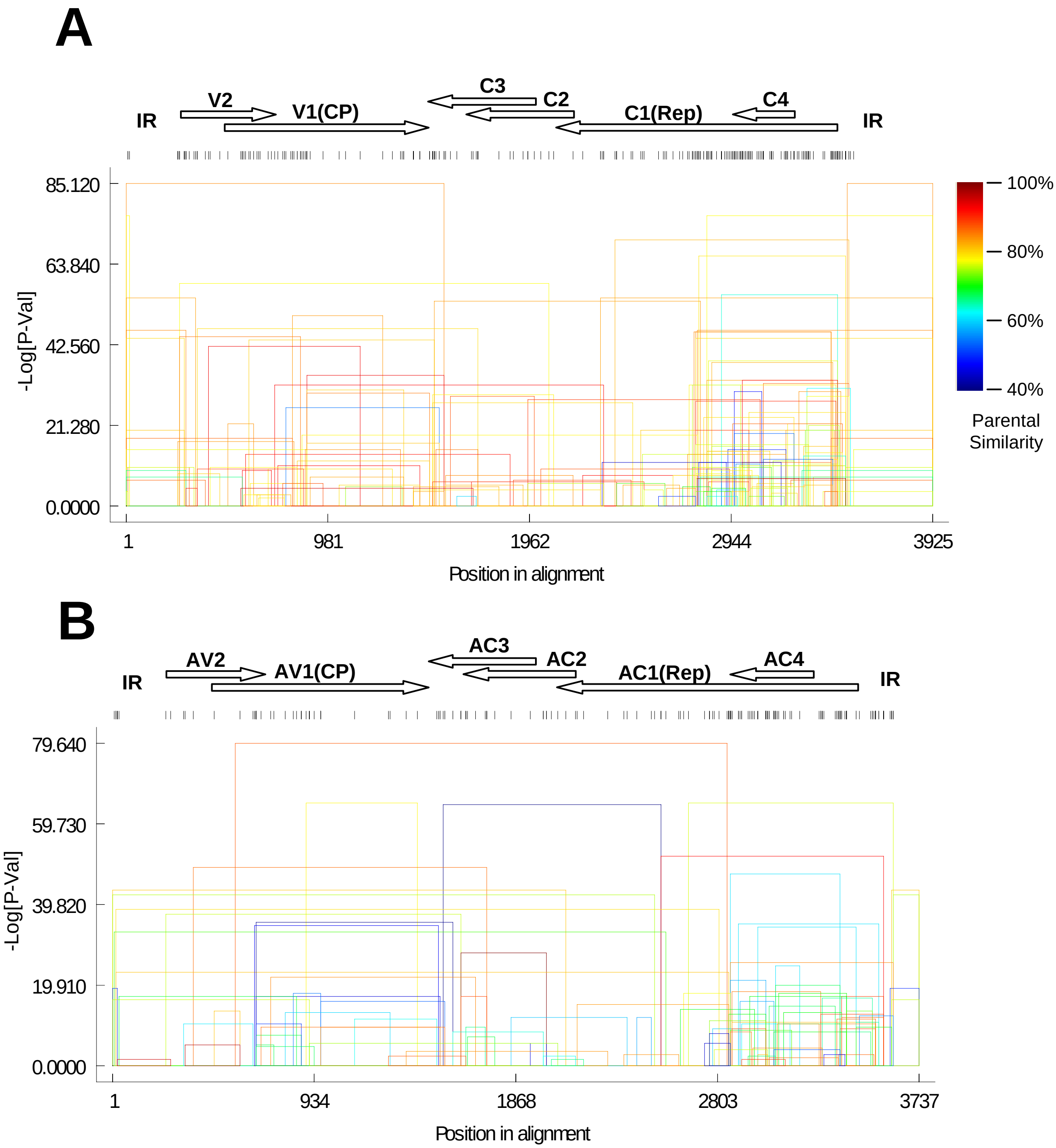

Supplementary figure 1. Schematic representations of individual recombination events detectable within the genomes of monopartite begomoviruses (A) and the DNA-A components of bipartite begomoviruses (B). Each rectangle represents one recombination event with vertical lines delimiting the tract of sequence transferred during the event. Whereas the heights of the vertical lines indicate the statistical certainty with which individual events were detected (in general greater statistical certaintly correlates with greater accuracy of breakpoint estimation), the colours of boxes reflect the approximate degree of parental relatedness at the time when recombination events occured.
