# Supplementary material for: Avoidance of Protein Fold Disruption in Natural Virus Recombinants
Source: PLoS Pathog. 2007 Nov 30;3(11):e181. doi: 10.1371/journal.ppat.0030181 (PMC2092379; doi:10.1371/journal.ppat.0030181)
Supplement: Supplementary file 2 [file ppat.0030181.sg002.pdf]

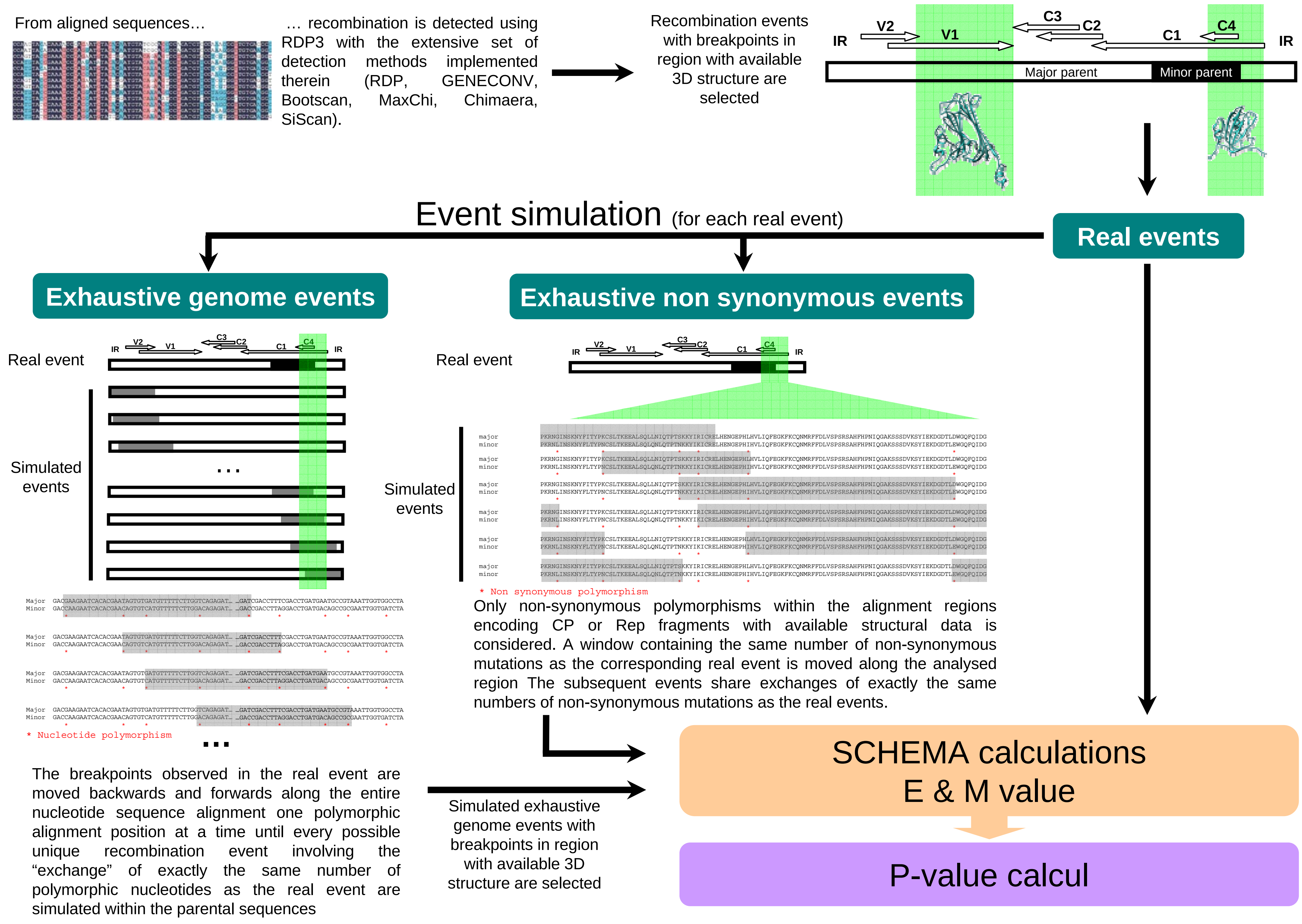

From aligned sequences…
… recombination is detected using RDP3 with the extensive set of detection methods implemented therein (RDP, GENECONV, Bootscan, MaxChi, Chimaera, SiScan).
Recombination events with breakpoints in region with available 3D structure are selected
IR
V2
V1
C3
C2
C1
C4
IR
Major parent
Minor parent
Event simulation (for each real event)
Real events
Exhaustive genome events
Exhaustive non synonymous events
Real event
Simulated events
Real event
Simulated events
major
minor
* Non synonymous polymorphism
Major
Minor
* Nucleotide polymorphism
Only non-synonymous polymorphisms within the alignment regions encoding CP or Rep fragments with available structural data is considered. A window containing the same number of non-synonymous mutations as the corresponding real event is moved along the analysed region The subsequent events share exchanges of exactly the same numbers of non-synonymous mutations as the real events.
The breakpoints observed in the real event are moved backwards and forwards along the entire nucleotide sequence alignment one polymorphic alignment position at a time until every possible unique recombination event involving the “exchange” of exactly the same number of polymorphic nucleotides as the real event are simulated within the parental sequences
Simulated exhaustive genome events with breakpoints in region with available 3D structure are selected
SCHEMA calculations
E & M value
P-value calcul
